# Serum from the Human Fracture Hematoma Contains a Potent Inducer of Neutrophil Chemotaxis

**DOI:** 10.1007/s10753-018-0760-4

**Published:** 2018-03-06

**Authors:** Okan W. Bastian, Mikolaj H. Mrozek, Marco Raaben, Luke P. H. Leenen, Leo Koenderman, Taco J. Blokhuis

**Affiliations:** 1 Department of Traumatology, University Medical Center Utrecht, Utrecht, The Netherlands; 2 Department of Respiratory Medicine and Laboratory of Translational Immunology (LTI), University Medical Center Utrecht, Utrecht, The Netherlands; 3 Department of Traumatology, Maastricht University Medical Center, Maastricht, The Netherlands

**Keywords:** fracture, neutrophil, CHIPS, CHIPSΔ1F, C5aR, CXCR1/2

## Abstract

A controlled local inflammatory response is essential for adequate fracture healing. However, the current literature suggests that local and systemic hyper-inflammatory conditions after major trauma induce increased influx of neutrophils into the fracture hematoma (FH) and impair bone regeneration. Inhibiting neutrophil chemotaxis towards the FH without compromising the hosts’ defense may therefore be a target of future therapies that prevent impairment of fracture healing after major trauma. We investigated whether chemotaxis of neutrophils towards the FH could be studied *in vitro*. Moreover, we determined whether chemotaxis of neutrophils towards the FH was mediated by the CXCR1, CXCR2, FPR, and C5aR receptors. Human FHs were isolated during an open reduction internal fixation (ORIF) procedure within 3 days after trauma and spun down to obtain the fracture hematoma serum. Neutrophil migration towards the FH was studied using Ibidi™ Chemotaxis^3D^ μ-Slides and image analysis of individual neutrophil tracks was performed. Our study showed that the human FH induces significant neutrophil chemotaxis, which was not affected by blocking CXCR1 and CXCR2. In contrast, neutrophil chemotaxis towards the FH was significantly inhibited by chemotaxis inhibitory protein of *Staphylococcus aureus* (CHIPS), which blocks FPR and C5aR. Blocking only C5aR with CHIPSΔ1F also significantly inhibited neutrophil chemotaxis towards the FH. Our finding that neutrophil chemotaxis towards the human FH can be blocked *in vitro* using CHIPS may aid the development of therapies that prevent impairment of fracture healing after major trauma.

## INTRODUCTION

Fracture healing starts with an inflammatory phase during which leukocytes infiltrate the blood collection surrounding the fracture site [1, 2]. Animal studies suggest that this blood collection, which is generally referred to as fracture hematoma (FH), forms a reservoir of essential factors and cells that regulate downstream processes of bone repair. This is illustrated by the finding that transplantation of the FH into muscle tissue induced ectopic bone formation and angiogenesis in animal models [3, 4]. Moreover, removal or repetitive irrigation of the FH impaired fracture healing in rats [5, 6].

Although controlled local inflammation is essential for adequate fracture healing [7], several animal studies have also shown that both local and systemic “hyper-inflammatory” conditions impair bone regeneration. For instance, injection of beta-glucan into the fracture site induces local hyper-inflammation and impairs fracture healing in rats [8]. Moreover, intraperitoneal injection of lipopolysaccharides in rats induces systemic inflammation and negatively affects the outcome of bone repair [9]. In addition, blunt chest injury, which is a model of trauma-induced systemic inflammation, also impairs fracture healing in rats [10].

It is well known that severely injured patients have an increased risk of developing impaired fracture healing [11, 12]. This not only has a significant impact on quality of life, but also carries a substantial economical burden to society [13]. Based on the abovementioned animal studies, we hypothesized that the systemic immune response after major trauma contributes to the high incidence of impaired fracture healing in multitrauma patients [1, 11]. The underlying mechanism remains unclear. However, experimental studies suggest that major trauma pre-activates neutrophils and induces increased influx of neutrophils towards sites of inflammation, such as the fracture hematoma [10, 14, 15], and impairs bone healing.

Such a pathological role of neutrophils was supported by the finding that depletion of neutrophils improved the outcome of bone repair in rats [16, 17]. However, systemic depletion of neutrophils would significantly compromise the hosts’ defense against pathogens.

Therefore, we tried to identify neutrophil chemoattractants within the sterile FH that may be blocked in the future without affecting chemotaxis of neutrophils towards sites of infection. As a first step, we tested whether neutrophil chemotaxis towards the human FH could be studied *in vitro*. Furthermore, we explored whether neutrophil chemotaxis towards the FH is mediated by IL-8 receptors CXCR1 and CXCR2, formylated peptide receptors (FPR), and complement receptor C5aR.

## MATERIALS AND METHODS

### Isolation of Neutrophils

Blood from anonymous healthy donors was acquired from the blood bank “Mini Donor Dienst” of the University Medical Center Utrecht after written informed consent was obtained. Neutrophils were isolated from peripheral blood, as has been described previously [18] and is summarized here. Briefly, 9 ml of blood was drawn into a sterile vacuum container with sodium citrate as anti-coagulant. The blood was diluted 1:1 in phosphate-buffered saline (PBS) at room temperature. The diluted peripheral blood was pipetted onto 15 ml of Ficoll-Paque (Pharmacia, Uppsala, Sweden) and centrifuged for 20 min at 900*g*. After centrifugation, the plasma, Ficoll, and mononuclear fraction were removed. The remaining erythrocytes and granulocytes were resuspended in 50 ml isotonic ice-cold ammonium chloride solution (NH_4_Cl) containing 155 mM NH_4_Cl, 10 mM KHCO_3_, and 0.1 mM EDTA (pH 7.2) and incubated on ice for 20 min. Subsequently, the cell suspension was centrifuged, the supernatant was removed, and the cell pellet was resuspended in 20 ml of NH_4_Cl. After centrifugation, the cell pellet was resuspended in HEPES3+ (20 mM HEPES, 132 mM NaCl, 6.0 mM KCl, 1.0 mM MgSO_4_, 1.2 mM KH_2_PO_4_, supplemented with 5.0 mM glucose, 1.0 mM CaCl2, and 0.5% (*w*/*v*) human serum albumin) and centrifuged again. The supernatant was removed and the cell pellet was resuspended in HEPES3+. Cells were counted using the Cell-Dyn® 1800 (Abbott Laboratories, Abbot Park, IL, USA) and diluted in HEPES3+ to concentrations needed during the experimental conditions (3.2 × 10^6^ cells/ml) and stored on ice until further use.

### Isolation of Human Fracture Hematoma Serum

Human fracture hematomas (FHs) were isolated during open reduction internal fixation (ORIF) procedures within 3 days after trauma from patients with closed fractures and without relevant comorbidity and collected in sterile plastic containers. The blood clot was isolated from the fracture site, which is generally required during an ORIF procedure in order to allow adequate reduction of the fracture and placement of fixation materials. FH was deemed residual tissue and could therefore be collected without obtaining informed consent, unless the patient explicitly refused (opt-out method). This procedure is formalized in our hospital and therefore approval by our local ethics committee was not required. All samples were stripped of identifiers and fully anonymized. The serum of the FHs was obtained by centrifugation (5 min, 2300 rcf) of the FH within 1 h after isolation. The serum was aliquoted and stored at − 20 °C until further use. The FH sera of different donors were used for each experimental condition and these sera were not pooled. Thirty microliters of FH was used for each experiment. There was no significant difference in chemotaxis towards fresh or frozen FH. The variation in neutrophil response towards the FH of different donors is depicted in Fig. 2c.

### Chemotaxis Assay

The Ibidi™ Chemotaxis^3D^ μ-Slide was used to analyze neutrophil chemotaxis towards the FH serum in a three-dimensional, porous *in vitro* environment (IBIDI, Martinsried, Germany). Setup and data analysis of the Ibidi™ Chemotaxis^3D^ μ-Slide have been described previously by other authors [19]. The Ibidi™ Chemotaxis^3D^ μ-Slide is a chemotaxis chamber that enables the investigator to create time-lapse images and videos of cell migration.

**Fig. 1:**
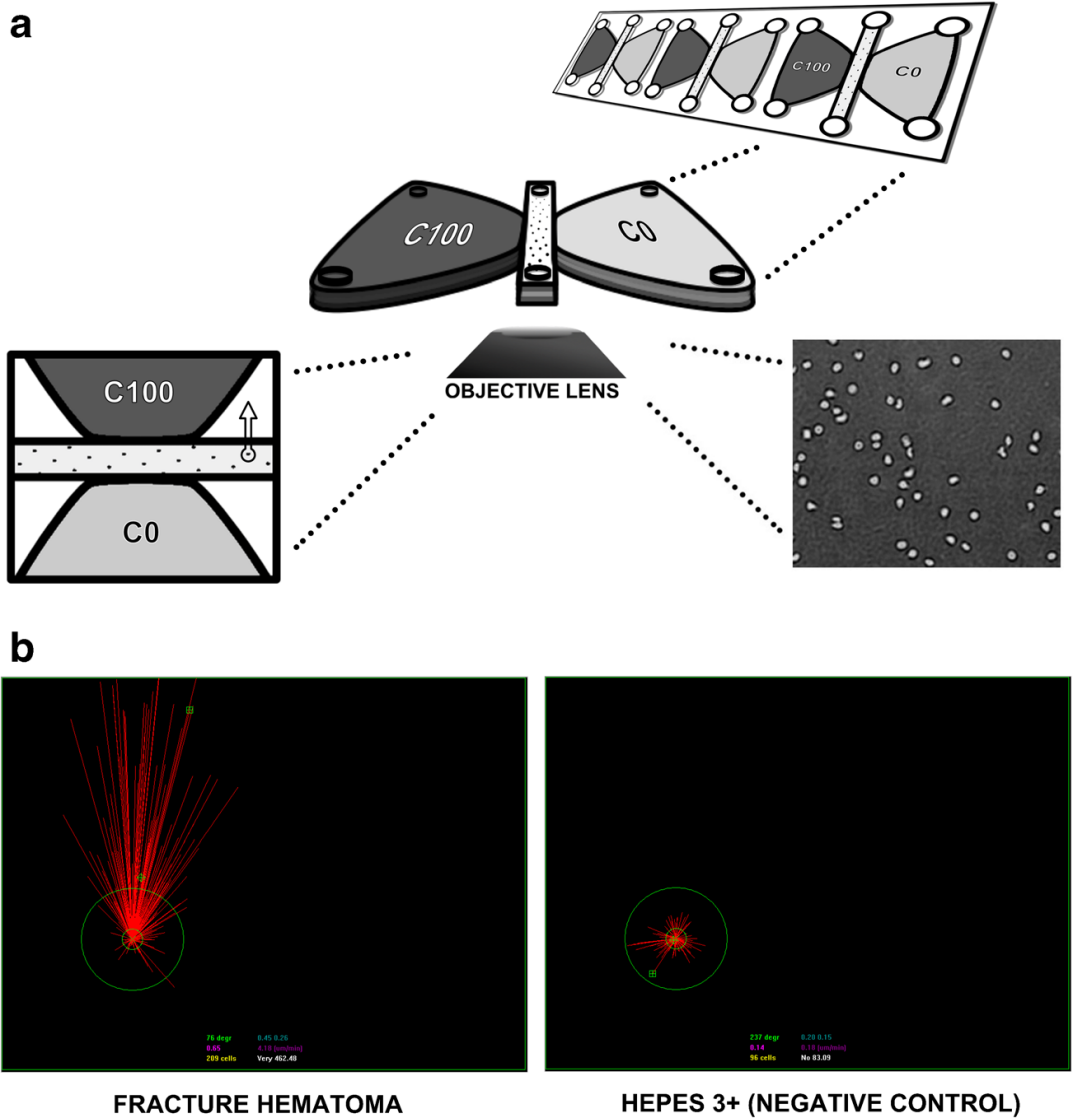
**a** Analysis of neutrophil chemotaxis towards the fracture hematoma using the Ibidi™ Chemotaxis^3D^ μ-Slide. A neutrophil/fibrinogen/thrombin suspension was injected into the observation area of the slide. After the fibrin gel solidified, HEPES3+ was injected into the C0 chamber. All experimental conditions were injected into the C100 chamber after which neutrophil chemotaxis was analyzed with time-lapse microscopy and cell tracking software. **b** Representative example of neutrophil migration towards the fracture hematoma and towards HEPES3+. The red lines are Euclidean distances, which are the shortest distances between each beginning and endpoint of all neutrophils that were analyzed. Vector speed was defined as the mean Euclidean distances of all neutrophils that were analyzed divided by imaging time.

Three microliters of ultra-pure human fibrinogen (25 mg/ml FIB3 obtained from Kordia, Leiden, the Netherlands) and 3.75 μl of thrombin (20 U/ml in PBS, purchased from Sigma, St. Louis, MO, USA) were added to the 30 μl of neutrophil suspension (final concentrations: fibrinogen 2.04 mg/ml; thrombin 2.04 U/ml; neutrophils 2.45 × 10^6^ cells/ml). Six microliters of this neutrophil/fibrinogen/thrombin suspension, containing approximately 1.5 × 10^4^ neutrophils, was pipetted into each center channel of Ibidi™ Chemotaxis^3D^ μ-Slide (observation area) using round tips according to the manufacturer’s protocol (Fig. 1a). This fibrin gel was allowed to solidify for 10 min at room temperature. HEPES3+ was pipetted into the right (C0) chamber and each experimental condition was pipetted into the left (C100) chamber (Fig. 1a).

A gradient of chemoattractants was rapidly established over the center channel (observation area). The slides were immediately placed in a pre-warmed microscopy chamber (37 °C, Heidolph Instruments inkubator 1000) onto an automated stage (Märzhäuser Wetzlar GmbH & Co., Wetzlar-Steindorf, Germany).

Time-lapse point revisiting microscopy (Quantimet 570C, DXMRE microscope, PL fluotar ×5 low power objective lens, Leica, Heidelberg, Germany) was used to track the movement of neutrophils through the fibrin gel. Sequences consisted of 100 images per spot with a maximum of 3 revisited spots. The time-lapse interval was typically 15–25 s. Consecutive images were converted into a movie using ImageJ (version 1.46r, Public Domain). OPTIMAS software (version 6.51, Media Cybernetics, Inc.) was used to derive trajectory plots and to quantify various parameters that describe chemotactic or chemokinetic responses which have been described previously [19]. Neutrophil chemotaxis was measured using mean vector speed, which is the Euclidean distance between starting point and end point of all neutrophils that were analyzed (Fig. 1b) divided by imaging time.

### Experimental Conditions

**Fig. 2:**
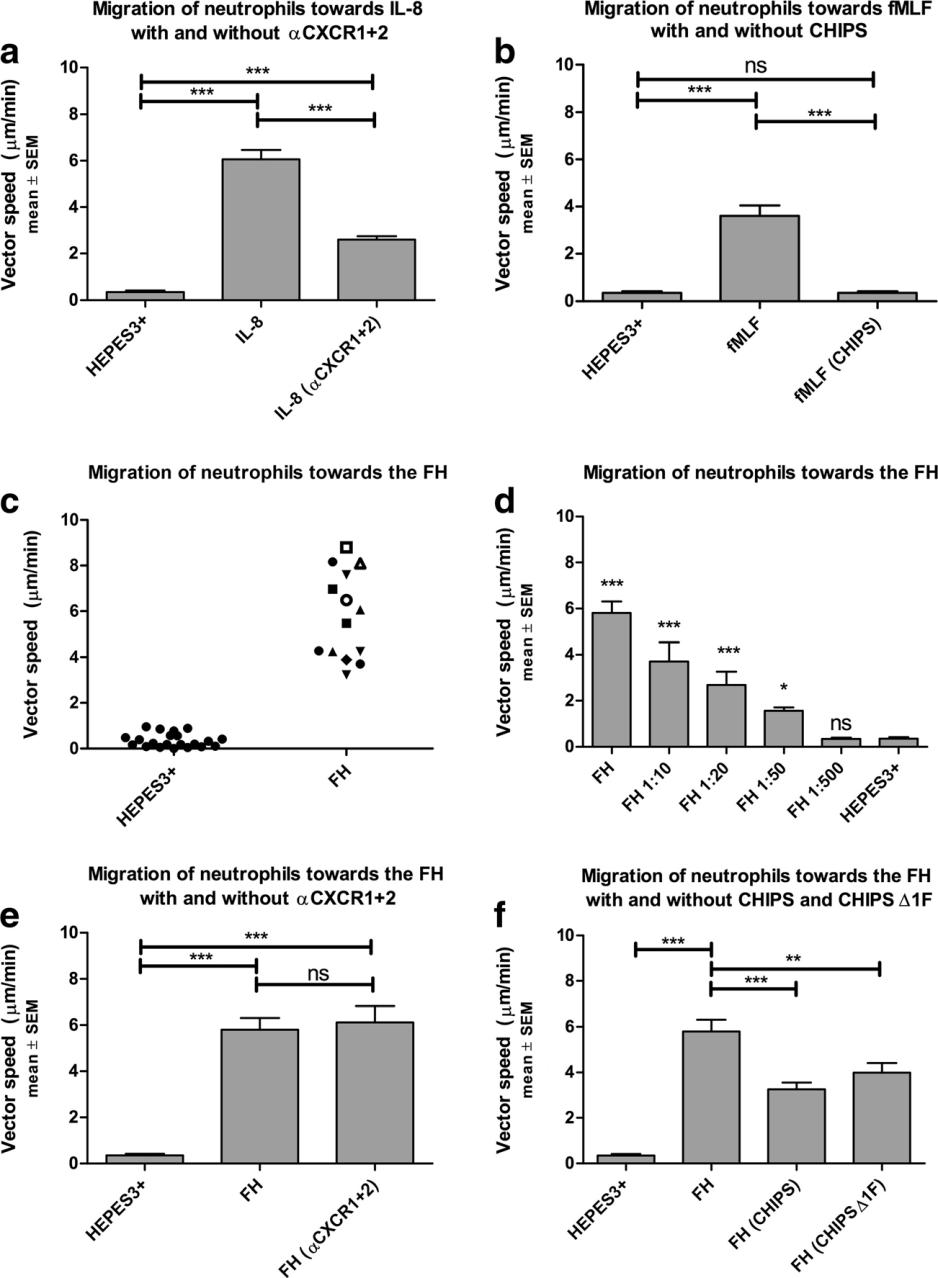
**a** Neutrophil chemotaxis towards IL-8 with and without blocking CXCR1 and CXCR2. IL-8 induced significant neutrophil chemotaxis compared to HEPES3+. Blocking CXCR1 and CXCR2 significantly inhibited migration towards IL-8. ***p < 0.001. **b** Migration of neutrophils through a 3D fibrin gel towards fMLF with and without blocking FPR with CHIPS. There was significant chemotaxis of neutrophils towards fMLF compared to the negative control HEPES3+. Blocking the FPR receptors with CHIPS significantly inhibited migration towards fMLF. ***p < 0.001. **c** Migration of neutrophils through a 3D fibrin gel towards the fracture hematoma (donor variation). Neutrophil migration towards the FH is depicted for each neutrophil/FH donor combination. Fourteen neutrophil donors were combined with 8 FH donors (14 neutrophil/FH donor combinations). A distinct icon is used to plot each FH donor. We were unable to find a significant difference in neutrophil migration towards the FH between different FH donors. **d** Migration of neutrophils through a 3D fibrin gel towards the fracture hematoma (dose response). Neutrophils significantly migrated towards the FH, even after diluting the FH 1:10, 1:20, and 1:50 in HEPES 3+. The 1:500 diluted FH did not induce significant neutrophil chemotaxis. ***p < 0.001 and *p < 0.05 compared to HEPES3+. **e** Neutrophil chemotaxis towards the fracture hematoma serum with and without blocking CXCR1 and CXCR2. Chemotaxis towards the FH was not significantly inhibited by blocking CXCR1 and CXCR2. ***p < 0.001. **f** Neutrophil chemotaxis towards the fracture hematoma with and without blocking C5aR and FPR with CHIPS and blocking C5aR with CHIPSΔ1F. CHIPS and CHIPSΔ1F significantly inhibited neutrophil chemotaxis towards the FH. ***p < 0.001, **p < 0.01.

*N*-Formyl-methionyl-leucyl-phenylalanine (fMLF) (Sigma-Aldrich, St. Louis, MO, USA), and recombinant human IL-8 (PeproTech EC Ltd., Rocky Hill, NJ, USA) were diluted in HEPES3+ (10^−7^ M and 50 ng/ml, respectively) and used as positive controls, since these factors are well-known chemoattractants for neutrophils [20, 21]. HEPES3+ was used as a negative control. CXCR1 and CXCR2 were simultaneously blocked on neutrophils using blocking antibodies αCXCR1 (Monoclonal Mouse IgG2A Clone # 42705, 500 μg/ml, R&D Systems®, Abingdon, UK) and αCXCR2 (Monoclonal Mouse IgG2A Clone # 48311 500 μg/ml, R&D Systems®, Abingdon, UK). Additionally, the C5aR and FPRs were simultaneously blocked using chemotaxis inhibitory protein of *Staphylococcus aureus* (CHIPS) which was donated and manufactured by the Department of Medical Microbiology, University Medical Center Utrecht, the Netherlands, as described by de Haas *et al*. [22, 23]. In addition, a CHIPS mutant lacking the first N-terminal amino acid was used (CHIPSΔ1F), which has impaired or absent FPR but still intact C5aR-blocking activity [24]. The isolated neutrophils were incubated with αCXCR1 and αCXCR2 or CHIPS and CHIPSΔ1F for 30 min on ice in 30 μl of solution (final concentrations: neutrophils 3.0 × 10^6^ cells/ml; αCXCR1/2 20 μg/ml; CHIPS and CHIPSΔ1F 10 μg/ml). After blocking the CXCR1 and CXCR2 receptors, neutrophil chemotaxis towards IL-8 and the FH was studied. After blocking the C5aR and FPR receptors with CHIPS, neutrophil chemotaxis towards fMLF and the FH was studied. Subsequently, neutrophil chemotaxis towards the FH was studied after blocking C5aR with CHIPSΔ1F. We did not use technical duplicates or triplicates with the same FH/neutrophil donor combinations analyzed at the same time point. Figure 2 therefore depicts the pooled data of single experiments with different FH/neutrophil donor combinations analyzed at different time points.

### Statistical Analysis

GraphPad Prism version 5.00 was used for all statistical analyses. All experimental conditions were compared using an ANOVA with a Bonferroni multiple comparison post hoc test. Multiple dilutions of the FH were compared to HEPES3+ using an ANOVA with Dunnett’s multiple comparison test. A *p* value < 0.05 was considered statistically significant. *p* values are described in Fig. 2 as * (< 0.05), ** (< 0.01), and *** (< 0.001).

## RESULTS

### Chemotaxis of Human Neutrophils Towards fMLF and IL-8

As a control study, we first determined whether neutrophil chemotaxis towards interleukin-8 (IL-8) and fMLF could be studied with the Ibidi™ Chemotaxis^3D^ μ-Slides, since these two factors are well-known neutrophil chemoattractants. Neutrophil chemotaxis towards HEPES3+ (negative control), IL-8, and fMLF is depicted in Fig. 2a, b, respectively. When compared to HEPES3+, there was a significant increase in migration towards IL-8 (mean: 0.4 *vs* 6.1 μm/min, *n* = 21 *vs* 8, *p* < 0.001) and fMLF (mean: 0.4 *vs* 3.6 μm/min, *n* = 21 *vs* 14, *p* < 0.001).

### Blocking Chemotaxis of Human Neutrophils Towards IL-8 and fMLF

Chemotaxis of neutrophils towards IL-8 is dependent on the CXCR1 and CXCR2 receptors. Blocking these two receptors induced a significant decrease in vector speed (mean: 6.1 *vs* 2.6 μm/min, *n* = 8 *vs* 4, *p* < 0.001 without and with blocking the CXCR1 and CXCR2 receptors) as depicted in Fig. 2a. CHIPS specifically binds to the formylated peptide receptors (FPRs) and C5a receptor (C5aR). Chemotaxis of neutrophils towards fMLF was significantly inhibited by CHIPS (mean: 3.6 *vs* 0.4 μm/min, *n* = 14 *vs* 12, *p* < 0.001, without and with CHIPS), as depicted in Fig. 2b.

### Chemotaxis of Neutrophils Towards the Human Fracture Hematoma

Neutrophils exhibited very potent chemotaxis towards the human fracture hematoma (FH) serum *in vitro*. The variation in neutrophil migration towards the FH for each neutrophil/FH donor combination (*n* = 14) is depicted in Fig. 2c. A dose response is depicted in Fig. 2d. The vector speed of neutrophils towards the FH was significantly greater when compared to migration towards HEPES3+ (mean: 5.8 *vs* 0.4 μm/min, *n* = 14 *vs* 21, *p* < 0.001, respectively). Neutrophil chemotaxis towards the FH remained significant when the FH was diluted in HEPES3+ 1:10 (mean: 3.7 *vs* 0.4 μm/min, *n* = 4 *vs n* = 21, *p* < 0.001), 1:20 (mean: 2.7 *vs* 0.4 μm/min, *n* = 5 *vs* 21, *p* < 0.001), and 1:50 (mean: 1.6 *vs* 0.4 μm/min, *n* = 8 *vs* 21, *p* < 0.05). When the FH was diluted 1:500, no significant chemotaxis could be observed (mean: 0.4 *vs* 0.4 μm/min, *n* = 6 *vs* 21).

### Chemotaxis of Neutrophils Towards the Fracture Hematoma After Blocking the CXCR1, CXCR2, FPR, and C5aR Receptors

Blocking the CXCR1 and CXCR2 receptors did not significantly affect neutrophil chemotaxis towards the FH (mean: 5.8 *vs* 6.1 μm/min, *n* = 14 *vs* 8, without and with blocking the CXCR1 and CXCR2 receptors). There was still significant chemotaxis towards the FH after blocking these receptors compared to HEPES3+ (mean: 6.1 *vs* 0.4 μm/min, *p* < 0.001) as depicted in Fig. 2e. CHIPS, which blocks FPR and C5aR, significantly inhibited neutrophil chemotaxis towards the FH (mean: 5.8 *vs* 3.3 μm/min, *n* = 14 *vs* 4, *p* < 0.001 without and with CHIPS, Fig. 2f). In addition, CHIPSΔ1F, which only blocks C5aR, also induced a significant decrease in neutrophil chemotaxis towards the FH (mean: 5.8 *vs* 4.0 μm/min, n = 14 *vs* 5, *p* < 0.01 without and with CHIPSΔ1F, Fig. 2f).

## DISCUSSION

The current literature suggests that increased influx of neutrophils into the fracture hematoma (FH) during hyper-inflammatory conditions impairs fracture healing after major trauma [1, 25]. Future therapies that inhibit influx of neutrophils into the FH without compromising the hosts’ defense against pathogens may therefore prevent impairment of bone healing in multitrauma patients. Our study shows that chemotaxis of neutrophils towards the FH can be studied *in vitro* with Ibidi™ Chemotaxis^3D^ μ-Slides. We found that serum from the human FH significantly induces neutrophil chemotaxis, which was not affected by blocking the CXCR1 and CXCR2 receptors (Fig. 2e). In contrast, CHIPS induced a significant decrease in neutrophil chemotaxis towards the human FH *in vitro* (Fig. 2f). CHIPS is an exoprotein produced by several strains of *S. aureus* and is a potent inhibitor of neutrophil and monocyte chemotaxis towards C5a and formylated peptides like fMLF [23]. It is known that tissue injury induces complement activation and release of C5a [15, 26], as well as release of formylated peptides from mitochondria into the circulation [27]. CHIPS exclusively binds directly to the C5aR and FPR1 and FPR2 receptors, thereby preventing their natural ligands from activating these receptors [23, 28]. We additionally used a CHIPS mutant lacking the first N-terminal amino acid (CHIPSΔ1F), which has impaired or absent FPR but still intact C5aR-blocking activity [24]. Our data shows that blocking C5aR with CHIPSΔ1F also significantly inhibits neutrophil chemotaxis towards the FH (Fig. 2f). Previous studies have shown that systemic antagonism of the C5aR improves fracture healing after major trauma in rats [15]. It is tempting to speculate that systemic C5aR antagonism prevents increased influx of neutrophils into the FH and thereby reduces the deleterious effect of major trauma on fracture healing.

In our *in vitro* experiments, we were unable to completely block neutrophil chemotaxis towards the FH using CHIPS or CHIPSΔ1F. One possible explanation for this effect is that the concentrations of blocking antibodies were insufficient to completely block all receptors. Also, several additional neutrophil chemoattractants may be present within the FH that do not exert their effect through CXCR1/2, FPR, or C5aR. Neutrophils possess several receptors that detect chemoattractants, such as chemokines, complement components, and several other chemotactic lipids and peptides [29]. Nineteen chemokine receptors have been identified so far, which include seven CXC receptors (CXCR1–7), ten CCR (CCR1–10), one CX_3_CR (CX_3_CR1), and one CR (XCR1) receptor [30]. Neutrophils are traditionally known to express only a very limited number of chemokine receptors and mainly express CXCR1 and CXCR2 in healthy individuals [31]. CXCR1 and CXCR2 are used by neutrophils to recognize N-terminal ELR (glutamic acid-leucine-arginine) motif-containing CXC chemokines. Human CXCR1 binds to CXCL8 (interleukin-8/IL-8) and CXCL6 (granulocyte chemotactic protein-2) [20, 29], as well as the ECM breakdown product N-acetyl PGP [32]. These three factors can also bind to CXCR2. However, CXCR2 is more promiscuous and binds different additional CXC chemokines, including CXCL1 (growth regulated oncogene-alpha/GRO-α), CXCL2 (GRO-β), CXCL3 (GRO-γ), CXCL5 (epithelial cell-derived neutrophil activating peptide-78/ ENA-78), and CXCL7 (neutrophil activating protein-2/GCP-2) [29]. Our study implies that these CXCR1 and CXCR2 ligands are not relevant in migration of neutrophils towards the FH *in vitro*. However, although neutrophils in healthy individuals mainly express CXCR1 and CXCR2 [31], it has been shown that infiltrated neutrophils from patients with chronic inflammatory lung diseases and rheumatoid arthritis express additional chemokine receptors on their surface, *i.e.*, CCR1, CCR2, CCR3, CCR5, CXCR3, and CXCR4 [31]. Moreover, major trauma induces the release of several neutrophil subsets into the peripheral circulation, including young banded neutrophils and hyper-segmented neutrophils, which exhibit different properties and receptor expressions compared to mature neutrophils from healthy individuals [33]. Future studies may focus on the role of these neutrophil subsets in fracture healing and determine whether neutrophils within the FH express other chemokine receptors compared to neutrophils isolated from peripheral blood of healthy donors.

Another chemotactic factor for neutrophils is leukotriene B4 (LTB4), which is recognized by a high-affinity receptor (BLT1) and a low-affinity receptor (BLT2) [34]. Animal studies have shown that LTB4 mediates neutrophil influx after experimental spinal cord injury [35]. It is tempting to speculate that LTB4 also mediates neutrophil influx into other types of sterile tissue injury, such as bone injury. An additional chemoattractant for neutrophils is platelet-activating factor (PAF), which is a phospholipid that is bound by the PAF receptor (PAFR) [36]. Little is known about the role of PAF in tissue injury although animal studies did show that inactivation of PAF by PAF acetylhydrolase significantly decreased neutrophil influx in a rabbit model of myocardial ischemia/reperfusion injury [37]. Future studies should investigate to which extent the abovementioned factors are also relevant in chemotaxis of neutrophils towards the FH.

In summary, our study shows that chemotaxis of neutrophils towards the FH can be studied *in vitro* with Ibidi™ Chemotaxis^3D^ μ-Slides. We found that serum from the human FH significantly induces chemotaxis, which was not affected by blocking CXCR1 and CXCR2. In contrast, CHIPS and CHIPSΔ1F, which blocks C5aR, induced a significant decrease in chemotaxis of neutrophils towards the FH. These findings may aid the development of therapies that prevent impairment of fracture healing after major trauma.
